# Sufentanil Inhibits the Proliferation and Metastasis of Esophageal Cancer by Inhibiting the NF-*κ*B and Snail Signaling Pathways

**DOI:** 10.1155/2021/7586100

**Published:** 2021-12-06

**Authors:** Huiyan Tang, Chao Li, Yongsheng Wang, Liqiang Deng

**Affiliations:** ^1^Department of Oncology, XinTai People's Hospital, Taian, Shandong 271200, China; ^2^Department of Thoracic Surgery, Rizhao Central Hospital, Rizhao, Shandong 276800, China; ^3^Department of Thoracic Surgery, Gaotang County People's Hospital, Liaocheng, Shandong 252800, China; ^4^Department of Anesthesiology, Maternal and Child Healthcare Hospital of Shandong Province, Ji'nan, Shandong 250014, China

## Abstract

Sufentanil is a *μ*-opioid receptor agonist, widely used in intraoperative and postoperative analgesia of esophageal cancer. This study investigated the effects of sufentanil on the proliferation, invasion, and metastasis of esophageal carcinoma cells and its molecular mechanisms. Human esophageal carcinoma cells CaES-17 and Eca-109 were cultured in vitro. Different concentrations of sufentanil (1 and 10 *μ*mol/L) were added to the experimental group. MTT was used to detect the proliferative activity of esophageal carcinoma cells. The migration ability of esophageal carcinoma cells was measured by the scratch test. Transwell was used to detect the invasive ability of esophageal carcinoma cells. The EMT marker expression was detected by qPCR. Meanwhile, effects of sufentanil on NF-*κ*B and Snail expression and nucleation were evaluated. Establish a subcutaneous xenograft tumor model of nude mice with esophageal carcinoma cells and evaluate the antitumor effect of sufentanil. Sufentanil can inhibit the proliferation, invasion, and migration of CaES-17 and Eca-109 cells and has a dose-dependent relationship. The molecular mechanism showed that sufentanil could upregulate the expression of E-cadherin and inhibit the expression of vimentin. Sufentanil can inhibit the expression of NF-*κ*B and Snail, as well as the nuclear expression of NF-*κ*B and Snail. Xenograft tumor model results showed that sufentanil could inhibit tumor proliferation and NF-*κ*B and Snail expression in tumor tissues of nude mice. Sufentanil inhibits esophageal cancer epithelial-mesenchymal transition (EMT) by acting on NF-*κ*B and Snail signaling pathways to inhibit proliferation and metastasis of esophageal cancer.

## 1. Introduction

Esophageal cancer is malignant tumors of the digestive tract, ranking the 6^th^ in the world [1–4]. Lymph node metastasis of esophageal carcinoma is one of the important prognostic factors [5]. Therefore, there is need to clarify the molecular mechanism of esophageal cancer metastasis and to search for drugs that can inhibit the proliferation and metastasis of esophageal cancer [6, 7]. Tumor surgery is the main treatment for patients with solid tumors [8]. During the perioperative period, many factors may influence the circulation of tumor cells, thus increasing the probability of postoperative metastasis and recurrence of tumor patients [9]. Opioids are one of the essential drugs for perioperative analgesia in cancer patients, which may also affect the growth, proliferation, and metastasis of tumor cells through various mechanisms. However, the effect of opioid analgesics on the biological behavior of tumor cells remains unclear and needs further study.

Sufentanil is a newly synthesized *μ*-opioid receptor agonist [10–12]. Sufentanil is widely used in all kinds of operation and postoperative analgesia because of its strong analgesic effect, long duration, and fewer side effects than morphine. However, the effect of sufentanil on the biological behavior of esophageal cancer cells is rarely reported [13, 14]. At present, most studies on the effects of opioids on tumor cells are based on morphine, and there are few literatures on the effects and mechanism of sufentanil on esophageal cancer cells [15, 16].

NF-*κ*B is a family of transcriptional regulators with multidirectional regulation [17–19]. NF-*κ*B is closely related to the occurrence and development of tumors. Snail is a transcription factor which plays an important role in the process of epithelial-mesenchymal transformation [20, 21]. EMT is an important mechanism for many tumor cells, including esophageal cancer, to acquire the ability of invasion and metastasis [22, 23].

In this study, we observed the effects of sufentanil on the proliferation activity, invasion, and metastasis of esophageal carcinoma cells, in order to provide theoretical and experimental basis for the application of sufentanil in tumor therapy.

## 2. Methods

### 2.1. Cell Culture

Human esophageal carcinoma cells (CaES-17 and Eca-109) were purchased from American Type Culture Collection (ATCC, Manassas, VA, USA). The cells were cultured in the RPMI-1640 medium containing 10% fetal bovine serum (Gibco, Life Technologies, Rockville, MA, USA). In addition, the medium contains penicillin (100 U/mL) and streptomycin (100 U/mL) (Hycult, Life Technologies, Rockville, MA, USA). It was cultured in an incubator at 37°C with a volume fraction of 5% CO_2_ and a relative humidity of 90%. Cell growth was observed regularly. Subculture was performed after digestion with 0.25% trypsin every 2-3 days. All the cells used in the experiment were in the logarithmic growth stage.

### 2.2. Transwell Experiment

Add 5 × 10^5^ cells to the superior compartment (Millipore, USA). The lower compartment was 20% FBS with the RPMI-1640 medium, 500 *μ*L/well. After 24 h of culture, the cells were fixed and stained when they migrated to the bottom and lateral sides of the membrane. Three suitable regions were randomly selected for cell counting under a light microscope. Calculate the mean and take a picture. All the experiments were repeated at least three times.

### 2.3. Proliferation Experiment

The cells were inoculated in 96-well plates at a density of 5 × 10^4^/mL. 100 *μ*L cell suspension was added to each well. Blank pores are free of cells. Incubate overnight in a 5% CO_2_ incubator. After the cells were completely attached to the wall, the supernatant was carefully sucked from the 96-well plate. In the experimental group, the RPMI-1640 culture medium containing different concentrations of sufentanil (1, 10 *μ*mol/L) (Sigma-Aldrich, St. Louis, MO, USA) was added to each well, 200 *μ*L. The negative control group and the blank control group only added the culture medium 200 *μ*L. Six parallel control holes were set for each group. After incubation for 48 h, 5 mg/mL MTT 20 *μ*L was added to each well for 4 h. Carefully suck off the supernatant. DMSO 150 *μ*L (Sigma-Aldrich, St. Louis, MO, USA) is added per well. Shake the shaker gently for 10 min. After the purple particle was fully dissolved, the wavelength of the purple particle was determined at 570 nm by a microplate reader (MultiskanEX, Lab systems, Helsinki, Finland). Determine the absorbance (OD) value of each well.

### 2.4. Immunofluorescence

All treated esophageal cancer cells were cultured on 18 mm cover glass for 24 h. The cells were completely adherent to the wall and fixed with 4% paraformaldehyde. After 3% BSA was blocked, primary antibodies including E-cadherin (1 : 1000 dilution; Abcam, Cambridge, MA, USA) and vimentin (1 : 1000 dilution; Abcam, Cambridge, MA, USA) were added. Incubate at 4°C overnight. After washing with PBS, the cells were incubated with Alexa Fluor 488 or Alexa Fluor 594 secondary antibody (1 : 500 dilution). The nuclei were stained with 4,6-diamidine-2-phenyl indoles (DAPI). Then, observe the slide under the FV-1000 laser scanning confocal microscope (Olympus, Japan).

### 2.5. qPCR

Prepared tissues or cell samples were extracted by TRIzol. By using a reverse transcription kit (TaKaRa, Japan), 2 *μ*g RNA was reverse-transcribed into cDNA. Dilute the primer to 100 nmol/ml. PCR reaction parameters were predenaturation at 94°C for 5 min, denaturation at 94°C for 30 s, annealing for 45 s, extension at 72°C for 1 min, and extension at 72°C for 10 min after 25 cycles. With SYBR green fluorescent dye (Japan TaKaRa) configuration, the real-time quantitative fluorescence polymerase chain reaction system (Applied Biosystems, Foster City, CA, USA) was used for quantitative fluorescence analysis. GAPDH was used as the internal reference to analyze the relative expression level of each gene. NF-*κ*B forward: 5′-CGAGAGGAGCACAGATACCAC-3′, reverse: 5′-CGCTTCTTCTTCACACACTGGATT-3′. Snail1 forward: 5′- CCTCCCTGTCAGATGAGGAC-3′, reverse: 5′- CCAGGCTGAGGTATTCCTTG-3′. GAPDH forward: 5′-CGGGAAACTGTGGCGTGAT-3′, reverse: 5′ AGTGGGTGTCGCTGTTGAAGT-3′. Upstream primers of E-cadherin: 5′-CGCATTGCCACATACA-3', downstream primers: 5′-CGT-TAGCCTCGTTCTCA-3′. Vimentin upstream primer: 5′-CGCTTCGCCAACTACAT-3′, downstream primer: 5′-AGGG-CATCCACTACCACAG-3′. 2^−△△Ct^ was used to calculate the relative expression of target genes in each sample. The mean and standard deviation of three duplicate holes was calculated.

### 2.6. Plate Clone Formation Experiment

Each group of cells in the logarithmic growth phase was selected. Each cell was digested with 0.25% trypsin and beaten into individual cells. 500 cells were placed in each well of the 6-well plate. The cells were evenly dispersed and placed in a cell culture box at 37°C with 5% CO_2_ for 2 weeks. Discard the supernatant and carefully soak it twice with PBS. The cells were fixed with 5 mL 4% paraformaldehyde for 15 min. Then, the fixed solution was removed, and 0.1% crystal violet staining solution was added for 30 min. The number of clones with more than 10 cells was counted under a microscope (4x).

### 2.7. Xenograft Tumor Model

Esophageal cancer cells at the logarithmic growth stage were collected. The cell concentration was adjusted to 5 × 10^7^/mL with 0.9% sodium chloride solution. Nude mice were subcutaneously injected with 0.2 ml each. About 1 cm × 1 cm in size, the tumor tissue was removed as the tumor source of subcutaneous inhibition. The tumor tissue was cut into small tumor blocks of 1 mm^3^ for later use. The nude mice were anesthetized, and a small incision was made under the skin with scissors. Small tumor blocks were implanted, and the incision was bonded with OB glue. After successful modeling, the patients were randomly divided into the sufentanil group and control group (0.9% sodium chloride solution) when the tumor grew to an average volume of about 100 mm^3^, six in each group, and continuous administration for 4 weeks. The experiment was approved by the Ethics Committee of the Maternal and Child Healthcare Hospital of Shandong Province.

### 2.8. Immunohistochemical

Tumor tissue was isolated and preserved in formalin. Prepare paraffin sections. The immunohistochemical two-step method (Thermo Fisher Scientific, Waltham, MA, USA) was used to detect and calculate the positive index of each section. At 37°C, the endogenous enzyme activity was blocked by incubation with 3% H_2_O_2_ for 20 min. Goat serum was added to block 15 min. The excess liquid was removed and NF-*κ*B (ab32360, Abcam, Cambridge, MA, USA) and Snail (ab216347, Abcam, Cambridge, MA, USA) antibodies at 1 : 100 were added. Incubate overnight at 4°C. On the second day, it was restored to room temperature, and biotin-labeled secondary antibody IgG working solution was added after washing with PBS. Incubate at 37°C for 15 min. Add horseradish-labeled streptomycin working solution. Incubate at 37°C for 15 min. DAB (benzidine) color rendering, color rendering at room temperature. The staining degree of the specimen was controlled under the microscope. After 20 s of redyeing with hematoxylin, rinse with water and return to blue. Dehydration transparent, sealing, photographing, and other processing. Image-Pro Plus 6.0 software was used to analyze each photo and obtain the positive cumulative optical density value (IOD) of each photo. The higher the IOD value was, the stronger the positive expression was.

### 2.9. Statistical Analysis

Statistical software SPSS 13.0 (SPSS Inc., Chicago, IL, USA) was used to process the experimental data. All data were expressed as mean ± standard deviation. The comparison between the two groups was statistically analyzed by the *t* test. One-way ANOVA was used for comparison between groups. *P* < 0.05 was considered statistically significant.

## 3. Results

### 3.1. Sufentanil Reduces Cell Activity and Inhibits Cell Migration and Invasion in Human Esophageal Carcinoma Cell Lines

**Figure 1:**
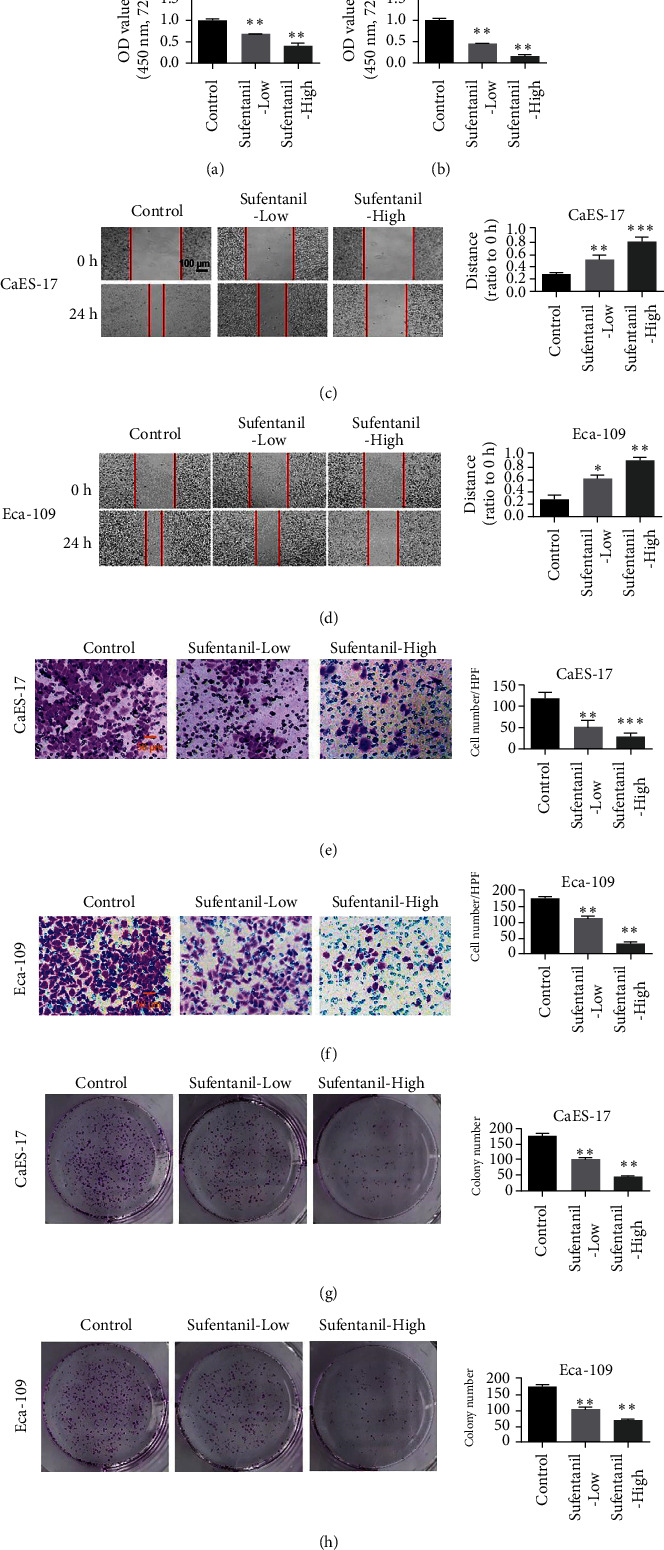
Sufentanil inhibits the proliferation, invasion, and migration of human esophageal carcinoma cells. (a) Proliferation ability of CaES-17 cells detected by MTT assay. (b) MTT assay used to detect the proliferation ability of Eca-109 cells. (c) The scratch test detecting migration ability of CaES-17 cells. (d) The scratch test detecting the migration ability of Eca-109 cells. (e) Transwell assay of CaES-17 cell invasion ability. (f) Transwell detecting the invasion ability of Eca-109 cells. (g) The clonogenesis experiments to evaluating the inhibitory effect of sufentanil on CaES-17 cell lines. (h) The clonogenesis experiments to evaluating the inhibitory effect of sufentanil on Eca-109 cell lines. ^∗^*P* < 0.05. ^∗∗^*P* < 0.01. Magnification: 200x.

In order to investigate the antitumor effects of sufentanil, we evaluated the inhibitory effects of sufentanil on the proliferation, migration, and invasion of human esophageal carcinoma cell lines by cell proliferation, cell scratch assay, and transwell. MTT assay was used to determine the effects of sufentanil treatment for 72 h on the cell viability of CaES-17 and Eca-109 cells. The experimental results showed that sufentanil inhibited the proliferation of CaES-17 and Eca-109 cells in a dose-dependent manner (Figures 1(a) and 1(b)). Next, we evaluated the ability of sufentanil to inhibit migration of CaES-17 and Eca-109 cells through wound-healing experiments. The results showed that compared with the control group, the migration distance of CaES-17 and Eca-109 cells in the sufentanil treatment group was significantly lower than that in the control group, with dose-dependent relationships (Figures 1(c) and 1(d)). Matrigel-coated transwell chambers were used to study whether sufentanil can inhibit the invasion of CaES-17 and Eca-109 cells. The experimental results showed that the number of cell invasion via the Matrigel-coated filter is reduced in a dose-dependent way compared with the control group (Figures 1(e) and 1(f)). We performed the clonogenesis experiments to further evaluate the inhibitory effect of sufentanil on esophageal cancer cell lines. The results showed that sufentanil could inhibit the clonogenesis of esophageal carcinoma cells (Figures 1(g) and 1(h)). Therefore, sufentanil can inhibit the proliferation, migration, and invasion of human esophageal carcinoma cell lines.

### 3.2. Sufentanil Reverses the EMT Biomarker of Human Esophageal Cancer Cell Line

**Figure 2:**
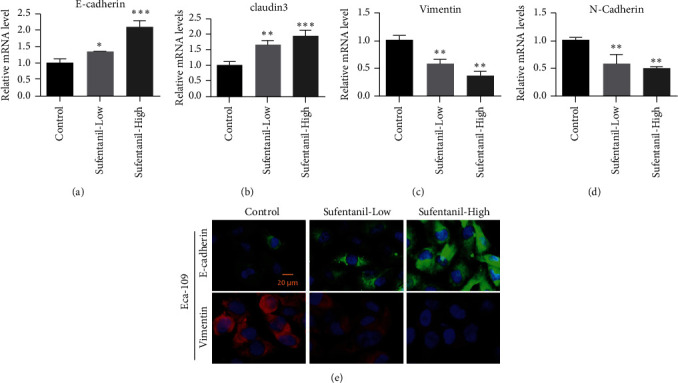
Sufentanil inhibiting EMT of esophageal carcinoma cells. (a)-(b) Expression of E-cadherin and claudin-3 detected by qPCR. (c)-(d) Vimentin and N-cadherin expression detected by qPCR. (e) Immunofluorescence staining used to detect the expression levels of E-cadherin and vimentin. ^∗^*P* < 0.05. ^∗∗^*P* < 0.01. Magnification: 400x.

To further investigate the molecular mechanism of sufentanil inhibiting human esophageal carcinoma cell lines, we investigated the effect of sufentanil on epithelial-mesenchymal transition in esophageal carcinoma cells. qPCR was used to detect the effects of sufentanil treatment on the levels of epithelial cell biomarkers (E-cadherin and claudin-3) and mesenchymal markers (vimentin and N-cadherin) in CaES-17 cells. The experimental results showed that the epithelial marker protein E-cadherin and claudin-3 levels of CaES-17 cells were elevated 24 h after treatment with different doses of sufentanil (Figures 2(a) and 2(b)). On the contrary, after sufentanil treatment, the expression levels of mesenchymal markers vimentin and N-cadherin decreased and showed dose-dependent relationships (Figures 2(c) and 2(d)). Furthermore, E-cadherin and vimentin were stained with immunofluorescence, and then, esophageal cancer cells were observed with the fluorescence microscope. The results of immunofluorescence assay were consistent with that of qPCR. Sufentanil can upregulate E-cadherin expression and inhibit vimentin expression (Figure 2(e)).

### 3.3. Sufentanil Inhibited NF-*κ*B Expression and Nuclear Entry in Human Esophageal Cancer

**Figure 3:**
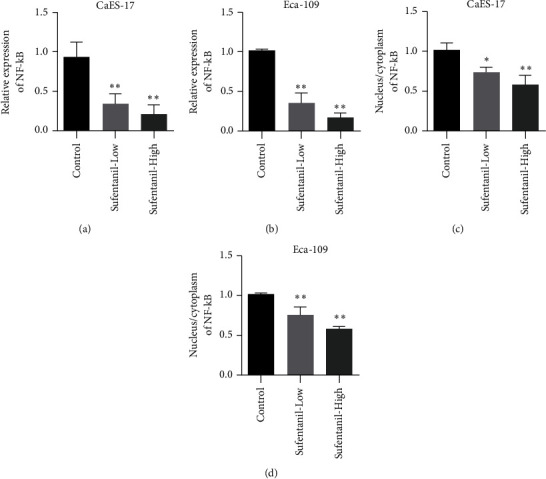
Sufentanil inhibiting NF-*κ*B in human esophageal carcinoma cells. (a) qPCR used to detect NF-*κ*B expression in CaES-17 cells. (b) qPCR used to detect NF-*κ*B expression in Eca-109 cells. (c) Immunofluorescence used to detect the nucleocytoplasmic ratio of NF-*κ*B in CaES-17 cells. (d) Changes of NF-*κ*B nucleoplasmic ratio in Eca-109 cells detected by immunofluorescence assay. ^∗^*P* < 0.05. ^∗∗^*P* < 0.01.

NF-*κ*B is an important signaling pathway molecule in tumor cells. We further examined the expression of NF-*κ*B in human esophageal carcinoma cell lines CaES-17 and Eca-109. Compared with the control group, sufentanil treatment inhibited the expression of NF-*κ*B (Figures 3(a) and 3(b)). NF-*κ*B needs to go into the nucleus to function as a transcription factor, so we also tested the content of NF-*κ*B in the nucleus. Sufentanil was shown to inhibit the nuclear NF-*κ*B content in CaES-17 and Eca-109 cells (Figures 3(c) and 3(d)).

### 3.4. Inhibitory Effect of Sufentanil on Human Esophageal Cancer

**Figure 4:**
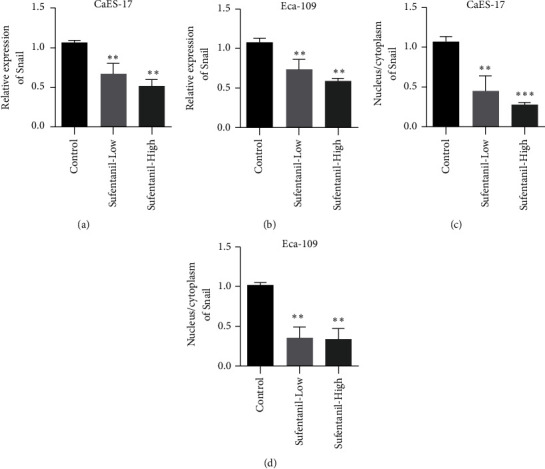
Sufentanil inhibiting human esophageal cancer cell Snail. (a) qPCR used to detect Snail expression in CaES-17 cells. (b) qPCR used to detect Snail expression in Eca-109 cells. (c) Immunofluorescence assay used to detect the cytoplasmic ratio of Snail in CaES-17 cells. (d) Immunofluorescence assay used to detect the cytoplasmic ratio of Snail in Eca-109 cells. ^∗∗^*P* < 0.01.

Snail is a member of the family of EMT-related transcription factors in tumor cells and a downstream regulator of NF-*κ*B. Snail plays an important role in EMT. qPCR results showed that sufentanil inhibited Snail expression in CaES-17 and Eca-109 cells (Figures 4(a) and 4(b)). In addition, in both CaES-17 and Eca-109 cell lines, Snail's proportion in the nucleus decreased (Figures 4(c) and 4(d)).

### 3.5. Sufentanil Has Been Shown to Have Antitumor Effects in Mouse Xenotransplantation Models

**Figure 5:**
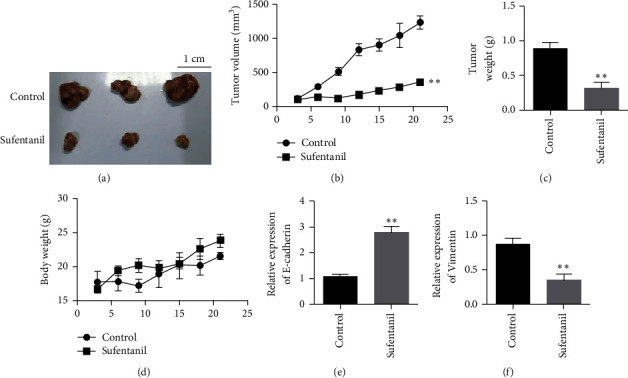
Animal experimental results showing that sufentanil inhibits human esophageal cancer cells. (a) Tumor images. (b) Tumor growth graph. (c) Tumor weight chart. (d) Statistical map of mouse weight. (e) The expression changes of E-cadherin in tumor tissues after sufentanil treatment. (f) The expression changes of vimentin in tumor tissues after sufentanil treatment. ^∗∗^*P* < 0.01.

Next, we investigated the effect of sufentanil on xenotransplantation of Eca-109 in nude mice. Compared with the control group, sufentanil treatment inhibited tumor growth (Figures 5(a) and 5(b)). Tumor weight test results also showed that sufentanil could inhibit the proliferation of subcutaneous transplanted tumors (Figure 5(c)). To evaluate the safety of sufentanil, we examined bodyweight in mice treated with sufentanil. The results showed that sufentanil had almost no effect on the weight of mice (Figure 5(d)). We detected the expression changes of E-cadherin and vimentin in tumor tissues after sufentanil treatment by qRT-PCR. The results showed that E-cadherin expression was upregulated, and vimentin expression was downregulated after sufentanil treatment (Figures 5(e) and 5(f)).

### 3.6. Sufentanil Inhibited the Expression of NF-*κ*B and Snail in Tumor Tissues

**Figure 6:**
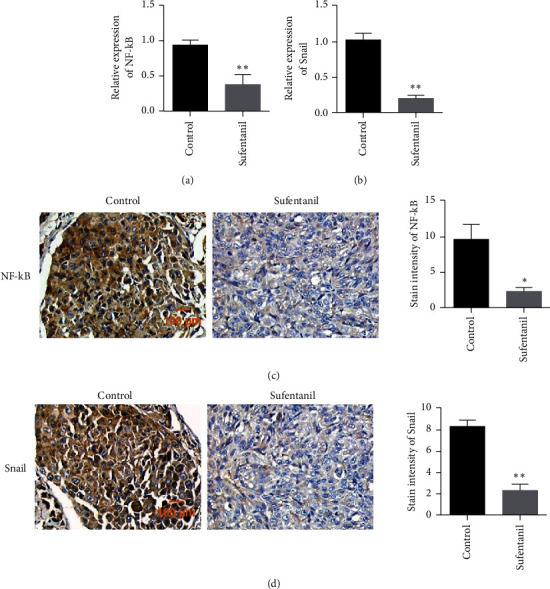
Changes in NF-*κ*B/Snail expression level detected in tumor tissues. (a) qPCR used to detect NF-*κ*B expression in tumor tissues. (b) qPCR used to detect Snail expression in tumor tissues. (c) Immunohistochemical detection of NF-*κ*B expression in tumor tissues. (d) Immunohistochemistry used to detect Snail expression in tumor tissues. ^∗^*P* < 0.05. ^∗∗^*P* < 0.01. Magnification: 200x.

Furthermore, the expression levels of NF-*κ*B and Snail in tumor tissues were detected by qPCR. The experimental results showed that compared with the control group, the expression of NF-*κ*B and Snail in tumor tissues after sufentanil treatment was significantly reduced (Figures 6(a) and 6(b)). Immunohistochemical staining of NF-*κ*B and Snail showed that sufentanil treatment reduced the expression levels of NF-*κ*B and Snail compared with untreated tumor tissue (Figures 6(c) and 6(d)).

## 4. Discussion

At present, opioid analgesics commonly used in clinical mainly include morphine, fentanyl, sufentanil, remifentanil, and so on. In recent years, many studies have shown that morphine and fentanyl can inhibit the growth, proliferation, and metastasis of tumors. For example, Tegeder et al. [24] observed morphine dose-dependent inhibition of adenocarcinoma cell proliferation in animal experiments. There are also reports that fentanyl can inhibit tumor growth, proliferation, and metastasis in mouse bone cancer models, and morphine can reduce tumor cell spread before and after operation [25–27].

Sufentanil, as a newly synthesized receptor agonist, has an analgesic potency 5–10 times that of fentanyl and 500–1000 times that of morphine [28–30]. Continuous administration has a more stable time-volume-dependent half-life than fentanyl. Sufentanil is less likely to cause drug accumulation and avoid serious complications such as respiratory depression. Sufentanil has fewer side effects than morphine and fentanyl and is often used to treat a variety of acute and chronic pain. The effects of sufentanil on proliferation, invasion, and metastasis of isolated esophageal carcinoma cells were studied in this study [29, 31, 32]. It was found that large doses of sufentanil could inhibit the proliferation of esophageal cancer cells and inhibit invasion and metastasis. This is consistent with the conclusion of Tegeder et al. [24] on the effect of morphine on the biological behavior of tumor cells.

There are several possible mechanisms of opioids to inhibit the biological behavior of tumor cells, including direct action on receptor or somatostatin receptor SSTR2 [33, 34]. By affecting the expression of cytokines, the immune function of patients is protected, and the growth of tumor cells is indirectly inhibited. Akural et al. [35] found that sufentanil preemptive analgesia can reduce the production of inflammatory cytokines IL-1 and IL-6. To protect the patient's immune function, inhibition of apoptosis plays an important role in the development and progression of most tumors. Many previous studies have suggested that morphine and fentanyl can both induce apoptosis of tumor cells [35, 36].

Current studies have found that TGF*β* [37], NF-kappaB [38], Ras/MAPK [39], and other mediated signaling pathways can induce Snail expression. Snail can act on the e-boxes connecting the motif 12 on the promoter, directly inhibiting the transcription of components of E-cadherin, claudins, occludins, plakophilin-2, and other epithelial adhesion sites, resulting in the disorder of intercellular adhesion. Snail can inhibit the expression of cytoskeleton components such as gelsolin, NHE-RF, ABLIM-1, and EPLIN and recombine the cytoskeleton [40]. Snail can also promote the expression of matrix metallo proteinases (MMP) [41] and degrade the matrix to promote cell invasion. In this study, sufentanil inhibited the proliferation of esophageal cancer cells, possibly by inhibiting the NF-*κ*B/Snail function [42–44].

In this study, MTT assay showed that sufentanil could significantly inhibit cell proliferation. In addition, animal results showed that sufentanil inhibited tumor volume. The results showed that sufentanil inhibited the proliferation of human esophageal carcinoma cells. RT-PCR results showed that the mRNA expression levels of NF-*κ*B and Snail genes decreased with the increase of drug concentration after treatment with sufentanil. Immunohistochemistry further confirmed the inhibitory effect of sufentanil on NF-*κ*B/Snail. These results suggest that sufentanil can downregulate the mRNA expression levels of NF-*κ*B and Snail genes in esophageal cancer cells, which may be one of the important mechanisms of inhibiting cancer, and the detailed mechanism of action needs to be further studied.

## 5. Conclusion

In summary, sufentanil was found to inhibit the proliferation, invasion, and metastasis of esophageal carcinoma cells in vitro and in vivo. Molecular mechanism studies have shown that sufentanil can achieve the anticancer effect by inhibiting NF-*κ*B/Snail. The elucidation of this study opens up a new way for the treatment of esophageal cancer, which is worthy of extensive clinical promotion.

## Data Availability

All data included in this study are available upon request by contact with the corresponding author.

## References

[1] Hinrichs C. S., van Way C. W. (2002). Esophageal cancer. *Current Surgery*.

[2] Japan Esophageal and Society (2009). Japanese classification of esophageal cancer, tenth edition: parts II and III. *Esophagus*.

[3] Aadam A. A., Abe S. (2018). Endoscopic submucosal dissection for superficial esophageal cancer. *Diseases of the Esophagus*.

[4] Wada H., Doki Y., Nishioka K. (2019). Clinical outcome of esophageal cancer patients with history of gastrectomy. *Journal of Surgical Oncology*.

[5] Corley D. A., Kerlikowske K., Verma R., Buffler P. (2003). Protective association of aspirin/NSAIDs and esophageal cancer: a systematic review and meta-analysis. *Gastroenterology*.

[6] Zhong W., Yang W., Qin Y. (2019). 6-Gingerol stabilized the p-VEGFR2/VE-cadherin/*β*-catenin/actin complex promotes microvessel normalization and suppresses tumor progression. *Journal of Experimental & Clinical Cancer Research*.

[7] Xi X., Liu N., Wang Q. (2019). ACT001, a novel PAI-1 inhibitor, exerts synergistic effects in combination with cisplatin by inhibiting PI3K/AKT pathway in glioma. *Cell Death & Disease*.

[8] Adelstein D. J., Rice T. W., Rybicki L. A. (2000). Does paclitaxel improve the chemoradiotherapy of locoregionally advanced esophageal cancer? a nonrandomized comparison with fluorouracil-based therapy. *Journal of Clinical Oncology*.

[9] Snyder G. L., Greenberg S. (2010). Effect of anaesthetic technique and other perioperative factors on cancer recurrence. *British Journal of Anaesthesia*.

[10] Zhao L., Li Y. (2020). Application of dexmedetomidine combined with sufentanil in colon cancer resection and its effect on immune and coagulation function of patients. *Oncology Letters*.

[11] Haiping D. (2018). *Effect of Different Concentrations of Dexmedetomidine Combined with Sufentanil on Cancer Postoperative Analgesia of Elderly Patients with Gastric Cancer in Laparoscopic Radical Resection*.

[12] Guo X., Yang X. (2018). *Effect of Sufentanil and Oxycodone on Immune Function of Laparoscopic Resection Colorectal Cancer*.

[13] Zhao J. H. (2011). *The Effects of Stress Reaction on Patients with Cancer of Stomach in Preemptive Analgesia by Sufentanil Combined Ropivacaine*.

[14] Wu W., Wei N., Jiang C. N., Cui S., Yuan J. (2014). Effects of sufentanil on human gastric cancer cell line SGC-7901 in vitro. *Central-European Journal of Immunology*.

[15] Mi K., Bonnyeo K., Ji Y., Yong S., Sung J., Haekeum K. (2005). The effects on urinary retention of epidural sufentanil in stomach cancer patients with gastrectomy. *Regional Anesthesia and Pain Medicine*.

[16] Mouedden M. E., Meert T. F. (2005). Evaluation of pain-related behavior, bone destruction and effectiveness of fentanyl, sufentanil, and morphine in a murine model of cancer pain. *Pharmacology Biochemistry and Behavior*.

[17] Yang Y., Li Y., Wang K., Wang Y., Yin W., Li L. (2013). P38/NF-*κ*B/snail pathway is involved in caffeic acid-induced inhibition of cancer stem cells-like properties and migratory capacity in malignant human keratinocyte. *PLoS One*.

[18] Chandra A., Nomura A., McGinn O. (2013). IL1B mediates NF-kB activity in pancreatic cancer cells. *Pancreas*.

[19] Wan Z., Pan H., Liu S. (2015). Downregulation of SNAIL sensitizes hepatocellular carcinoma cells to TRAIL-induced apoptosis by regulating the NF-Κb pathway. *Oncology Reports*.

[20] Hu Z., Liu X., Tang Z., Zhou Y., Qiao L. (2013). Possible regulatory role of snail in NF-*κ*B-mediated changes in E-cadherin in gastric cancer. *Oncology Reports*.

[21] Taki M., Abiko K., Baba T. (2018). Snail promotes ovarian cancer progression by recruiting myeloid-derived suppressor cells via CXCR2 ligand upregulation. *Nature Communications*.

[22] Feng M., Feng J., Chen W. (2016). Lipocalin2 suppresses metastasis of colorectal cancer by attenuating NF-*κ*B-dependent activation of snail and epithelial mesenchymal transition. *Molecular Cancer*.

[23] Huang T. A. O., Chen Z., Fang L. (2014). Curcumin inhibits LPS-induced EMT through down-regulating NF-Κb-snail signaling in breast cancer cells. *Journal of Xian Jiaotong University*.

[24] Tegeder I., Grösch S., Schmidtko A. (2003). G protein-independent G1 cell cycle block and apoptosis with morphine in adenocarcinoma cells: involvement of *p*53 phosphorylation. *Cancer Research*.

[25] Page G. G., McDonald J. S., Ben-Eliyahu S. (1998). Pre-operative versus postoperative administration of morphine: impact on the neuroendocrine, behavioural, and metastatic-enhancing effects of surgery. *British Journal of Anaesthesia*.

[26] El Mouedden M., Meert T. F. (2007). The impact of the opioids fentanyl and morphine on nociception and bone destruction in a murine model of bone cancer pain. *Pharmacology Biochemistry and Behavior*.

[27] Xi X., Chu Y., Liu N. (2019). Joint bioinformatics analysis of underlying potential functions €hsa-let-7b-5p and core genes in human glioma. *Journal of Translational Medicine*.

[28] Wu W., Wei N., Jiang C.-n., Cui S., Yuan J. (2014). Experimental immunology effects of sufentanil on human gastric cancer cell line SGC-7901 in vitro. *Central European Journal of Immunology*.

[29] Good P., Jackson K., Brumley D., Ashby M. (2009). Intranasal sufentanil for cancer-associated breakthrough pain. *Palliative Medicine*.

[30] Peng Z., Zhang Y., Guo J., Guo X., Feng Z. (2018). Patient-controlled intravenous analgesia for advanced cancer patients with pain: a retrospective series study. *Pain Research and Management*.

[31] Yang Y., Wu J., Li H. (2018). Prospective investigation of intravenous patient-controlled analgesia with hydromorphone or sufentanil: impact on mood, opioid adverse effects, and recovery. *BMC Anesthesiology*.

[32] Zhang W., Fang C., Li J. (2014). Single-dose, bilateral paravertebral block plus intravenous sufentanil analgesia in patients with esophageal cancer undergoing combined thoracoscopic-laparoscopic esophagectomy: a safe and effective alternative. *Journal of Cardiothoracic and Vascular Anesthesia*.

[33] Barg J., Belcheva M. M., Levy R. (1994). A monoclonal anti-idiotypic antibody to opioid receptors labels desipramine-induced opioid binding sites on rat C6 glioma cells and attenuates thymidine incorporation into DNA. *Glia*.

[34] Hatzoglou A., Kampa M., Castanas E. (2005). Opioid-somatostatin interactions in regulating cancer cell growth. *Frontiers in Bioscience*.

[35] Akural E. I., Salomäki T. E., Bloigu A. H. (2004). The effects of pre-emptive epidural sufentanil on human immune function. *Acta Anaesthesiologica Scandinavica*.

[36] Sueoka E., Sueoka N., Kai Y. (1998). Anticancer activity of morphine and its synthetic derivative, KT-90, mediated through apoptosis and inhibition of NF-*κ*B activation. *Biochemical and Biophysical Research Communications*.

[37] Peinado H., Quintanilla M., Cano A. (2003). Transforming growth factor *β*-1 induces snail transcription factor in epithelial cell lines. *Journal of Biological Chemistry*.

[38] Barberà M. J., Puig I., Domínguez D. (2004). Regulation of Snail transcription during epithelial to mesenchymal transition of tumor cells. *Oncogene*.

[39] Nieto M. A. (2002). The snail superfamily of zinc-finger transcription factors. *Nature Reviews Molecular Cell Biology*.

[40] Zhong W., Sun B., Gao W. (2018). Salvianolic acid A targeting the transgelin-actin complex to enhance vasoconstriction. *EBioMedicine*.

[41] Jordà M., Olmeda D., Vinyals A. (2005). Upregulation of MMP-9 in MDCK epithelial cell line in response to expression of the snail transcription factor. *Journal of Cell Science*.

[42] Lu Z., Li Y., Wang J. (2017). Long non-coding RNA NKILA inhibits migration and invasion of non-small cell lung cancer via NF-*κ*B/snail pathway. *Journal of Experimental & Clinical Cancer Research*.

[43] Bonavida B., Baritaki S. (2012). Inhibition of epithelial-to-mesenchymal transition (EMT) in cancer by nitric oxide: pivotal roles of nitrosylation of NF-*κ*B, YY1 and snail. *Forum on Immunopathological Diseases and Therapeutics*.

[44] Zhang (2015). Autocrine IL-8 promotes F-actin polymerization and mediate mesenchymal transition via ELMO1-NF-B-snail signaling in glioma. *Cancer Biology & Therapy*.

